# Mucosal delivery of CpG-ODN mimicking bacterial DNA via the intrapulmonary route induces systemic antimicrobial immune responses in neonatal chicks

**DOI:** 10.1038/s41598-020-61683-y

**Published:** 2020-03-24

**Authors:** Kalhari Goonewardene, Khawaja Ashfaque Ahmed, Thushari Gunawardana, Shelly Popowich, Shanika Kurukulasuriya, Ruwani Karunarathna, Ashish Gupta, Lisanework E. Ayalew, Betty Lockerbie, Marianna Foldvari, Suresh Tikoo, Philip Willson, Susantha Gomis

**Affiliations:** 1 Department of Veterinary Pathology, Western College of Veterinary Medicine, University of Saskatchewan, Saskatoon, SK S7N 5B4 Canada; 2 School of Pharmacy, University of Waterloo, 200 University Avenue West, Waterloo, ON N2L 3G1 Canada; 3 Vaccinology and Immunotherapy, School of Public Health, University of Saskatchewan, Saskatoon, SK S7N 5E3 Canada; 4 Canadian Centre for Health and Safety in Agriculture, University of Saskatchewan, Saskatoon, SK S7N 2Z4 Canada

**Keywords:** Toll-like receptors, Bacterial host response

## Abstract

The transition to antibiotic-free poultry production in the face of pathogenic threats is a very challenging task. We recently demonstrated that mucosal delivery of CpG-ODN alone by the intrapulmonary route (IPL) has potential as an effective alternative to antibiotics in neonatal chicks against *Escherichia coli* septicemia. How exactly mucosal delivery of CpG-ODN elicits, protective antibacterial immunity remained poorly understood. In this study, CpG-ODN or saline was delivered via the intrapulmonary route to day-old chicks (n = 80/group) using a compressor nebulizer in an acrylic chamber (1 mg/mL CpG-ODN for 15 minutes). In the first part of the study, two days after mucosal CpG-ODN delivery, 40 chicks from each group were challenged subcutaneously with 1 × 10^5^ cfu (n = 20) or 1 × 10^6^ cfu (n = 20) of *E. coli* and the mortality pattern was monitored for seven days. We found significantly higher survival, better clinical conditions and lower bacterial loads in chicks that received mucosal CpG-ODN. To explore the mechanisms behind this protective immunity, we first looked at the kinetics of the cytokine gene expression (three birds/ group/ time for 10 time-points) in the lungs and spleens. Multiplex gene analysis demonstrated a significant elevation of pro-inflammatory cytokine genes mRNA in the CpG-ODN group. Interleukin (IL)-1β robustly upregulated many folds in the lung after CpG-ODN delivery. Lipopolysaccharide-induced tumor necrosis factor (LITAF) and IL-18 showed expression for an extended period in the lungs. Anti-inflammatory cytokine IL-10 was upregulated in both lungs and spleen, whereas IL-4 showed upregulation in the lungs. To investigate the kinetics of immune enrichment in the lungs and spleens, we performed flow cytometry, histology, and immunohistochemistry at 24, 48 and 72 hrs after CpG-ODN delivery. CpG-ODN treated lungs showed a significant enrichment with monocytes/macrophages and CD4^+^ and CD8^+^ T-cell subsets. Macrophages in CpG-ODN treated group demonstrated mature phenotypes (higher CD40 and MHCII expression). Importantly, mucosal delivery of CpG-ODN via the intrapulmonary route significantly enriched immune compartment in the spleen as well, suggesting a systemic effect in neonatal chicks. Altogether, intrapulmonary delivery of aerosolized CpG-ODN orchestrates protective immunity against *E. coli* septicemia by not only enhancing mucosal immunity but also the systemic immune responses.

## Introduction

The emergence of antimicrobial resistant (AMR) strains of bacteria due to antimicrobial use (AMU) in animal production is a global problem. AMR is quintessentially a one-health issue. The Canadian chicken industry is implementing AMU reduction strategies to control AMR development. However, studies suggest that withdrawal of prophylactic antibiotic use led to a substantial increase in therapeutic antibiotic use in the poultry industry^1^. Broiler chickens are most susceptible to bacterial and viral infections during the first week of their life, and these infections adversely influence the remaining production cycle and growth of chickens^2,3^. As a result, the poultry industry is urgently looking for suitable alternatives to antibiotics for disease prevention. Over a decade, our group has been studying immunomodulation using synthetic oligodeoxynucleotides containing unmethylated cytosine-phosphodiester-guanine motifs (CpG-ODN) in chickens. We provided the first *in vivo* evidence for the standalone antimicrobial function of CpG-ODN in chicks against *Escherichia coli* and *Salmonella* infections using *in ovo* (embryo injection)^4,5^, intramuscular and subcutaneous^6,7^ routes of delivery.

CpG-ODNs are short, single-stranded DNA that can stimulate the immune system by acting as pathogen-associated molecular patterns (PAMPs)^8–10^. CpG-ODN initiates immune modulation by binding to specific Pattern Recognition Receptors (PRRs) called toll-like receptor (TLR)-9 (mammalian species) or TLR-21 (avian species) in antigen presenting cells (APCs) such as dendritic cells, macrophages, and B lymphocytes^11–14^. Functionally, both TLR-9 and TLR-21 are similar in CpG-ODN pattern recognition although there is species-specific variability in recognizing different contexts of nucleotide sequences in the CpG motifs^15^. Murine TLR-9 recognizes the GACGTT motifs whereas human TLR-9 and chicken TLR-21 recognizes the GTCGTT motifs comprising CpG-ODN^16^. Both human and avian CpG-ODN elicits similar cytokine induction and activation of immune cells leading to innate and adaptive immune responses^17–19^. Furthermore, the immune stimulatory ability of CpG-ODN can be potentiated by formulating it with nano particles^4,9,20,21^. A study conducted using a chicken macrophage cell line (HD11 cells) reported that CpG-ODN induced strong interleukin (IL)-6 and nitric oxide secretion while killing *Salmonella enteritidis* in the activated cells^22^. There is evidence that CpG-ODNs innate immune stimulation results in high nitric oxide production in monocytes, which may be directly associated with its ability to control microbial infections^23^. Other studies have reported that CpG-ODN stimulates the expression of interferon (IFN)-γ, IL-1β, IL-6, and IL-8 in spleens^24,25^. A study by our group showed that *in ovo*CpG-ODN injection in 18-days old embryonated eggs elicited a significantly higher expression of pro-inflammatory cytokines^26^.

We recently reported that mucosal delivery of aerosolized CpG-ODN micro-droplets via the intrapulmonary route can protect neonatal broiler chicks against lethal *E.coli* septicemia^27^. However, the mechanisms by which mucosal delivery of aerosolized CpG-ODN alone provides protection in chickens against *E.coli* septicemia are not completely understood. The present study was designed to gain greater insights into the antimicrobial protective mechanisms resulting from mucosal delivery of aerosolized CpG-ODN in neonatal chicks.

## Materials and methods

### CpG-ODN

The sequence of CpG-ODN used was 5′-TCGTCGTTGTCGTTTTGTCGTT-3′. It was free of endotoxin and produced with a phosphorothioate backbone (Operon Biotechnologies, Inc; Huntsville, AL, USA).

### Bacterial strain and its culture for the challenge

A field isolate of *E. coli* from a turkey with septicemia was used as the bacterial strain to challenge the birds. This *E. coli* belonged to serogroup O2, and it was non-hemolytic, serum-resistant, aerobactin-producing, with a K1 capsule and Type 1 pili. Aliquots of bacteria were stored at −80 °C in 50% brain–heart infusion broth (Difco, Detroit, MI) supplemented with 25% (w/v) glycerol (VWR Scientific, Inc., Montreal, Quebec). Bacterial culture was prepared as previously described^27^. Briefly, bacteria used for the challenge were cultured on 5% Columbia sheep blood agar plates for 18–24 hr at 37 °C. One colony was added to 100 mL of Luria broth in a 250 mL Erlenmeyer flask. The culture was grown at 37 °C for 13 hr with shaking at 150 rpm. Stationary phase culture contained approximately 1 × 10^9^ colony forming units (cfu) of bacteria per mL. The cultures were diluted in sterile saline, so the concentration of bacteria required for the challenge (1 × 10^5^ or 1 × 10^6^ cfu/bird) was obtained. Viable bacterial counts were determined by plating serial dilutions of the diluted culture in duplicate on 5% Columbia sheep blood agar plates, incubating for 18–24 hr at 37 °C; then counting the number of colonies.

### Chickens, animal housing and maintenance

This work was approved by the Animal Research Ethics Board, University of Saskatchewan and adhered to the guidelines of the Canadian Council on Animal Care. Hatching eggs (broiler strain Ross 308) were obtained from a commercial hatchery in Saskatchewan, Canada and incubated at the Animal Care Unit (ACU), Western College of Veterinary Medicine, University of Saskatchewan. Hatched chicks were allocated randomly into an animal isolation room at the ACU with wood shaving on the floor as litter. A tag with an identification number was placed on the neck to identify the groups. Water and commercial broiler ration were provided *ad libitum*. The animal room was ventilated with filtered, non-recirculated air at a rate of 10–12 changes/hr. In addition, air pressure differentials and strict sanitation were maintained in this isolation facility. Broilers were raised at 32 °C for the first week of life; after that the temperature was decreased 0.5 °C per day until a room temperature of 27.5 °C was reached. The light was provided for 24 hr/d during days 0 to 2 post-hatch. Darkness was introduced at 3 d post-hatch with 1 hr of dark added daily until 4 hr of darkness was achieved.

### *E.coli* challenge of neonatal chicks

The *E. coli* animal challenge studies were carried out following the procedure as described previously^27^. Briefly, 2 d post-treatment, birds were challenged subcutaneously in the neck with either 1 × 10^5^ or 1 × 10^6^ cfu of *E. coli*. Two doses of *E. Coli* were used in our study to simulate field conditions wherein all birds are not usually exposed to a consistent dose of *E. coli*. Birds were evaluated three times daily at the critical stage (until 3 d post-challenge) and twice thereafter up to 7 d post-challenge. Each bird was observed for their clinical presentation and a daily clinical score was assigned: 0 = normal; 0.5 = slightly abnormal appearance, slow to move; 1 = depressed, reluctant to move; 1.5 = reluctant to move, may take a drink and peck some; 2 = unable to stand or reach for food or water; and 3 = dead. Birds that received a clinical score of 2 were euthanized by cervical dislocation. At the end of the trial, each bird was given a cumulative clinical score (CCS) as a sum of daily clinical scores as previously described^7,27^. Chicks that were found dead or euthanized were subjected to immediate necropsy. On day 7 post-challenge, the remaining birds were humanely euthanized by cervical dislocation. Bacterial swabs were taken from the air sacs of dead and euthanized birds, and cultured on 5% Columbia sheep blood agar according to the quadrant streaking technique. In quadrant streaking method, bacterial reduction occurs as streaking moves clockwise from quadrant 1 to quadrant 4. A semi-quantitative estimate of *E. coli* isolation was conducted according to the growth on blood agar. Growth on these plates was recorded on a scale from 0 to 4+ , where 0 = no growth; few = less than 5 colonies; 1+ = growth of bacteria on quadrant 1 only; 2+ = growth of the bacteria on quadrants 1–2; 3+ = growth of bacteria on quadrants 1–3; and 4+ = growth of bacteria on quadrants 1–4^28^.

### Preparation of cells for flow cytometry

Preparation of cells and antibody staining for flow cytometry was done as previously described with slight modifications^29–32^. Briefly, whole lung and spleen were harvested from chicks at 24, 48 and 72 hr post CpG-ODN treatmentand processed for single cell preparation. Each whole spleen was gently pushed through a metal strainer by applying gentle pressure using a syringe plunger in ~3 mL of phosphate buffered saline (PBS) and cells were collected in a 15 mL centrifuge tube. The whole lung was manually chopped into small pieces using a surgical blade and incubated at 37 C for 30 min with ~1 mL of collagenase (1 mg/mL) dissolved in Dulbecco’s Modified Eagle Medium. After incubation, these tissues were similarly pushed through a metal strainer to obtain a single cell suspension and washed twice with PBS. Then, whole lung and spleen cells were incubated for 15–20 min with red blood cells lysis buffer. Following three washes with wash buffer (PBS containing 2% fetal bovine serum and 0.1% sodium azide), the whole lung and spleen cells from control and CpG-ODN groups were resuspended in equal volume of PBS (8 mL). Then, 1 mL of the suspension from control and CpG-ODN samples were used for antibody staining and analyzed by flow cytometry.

### Antibodies for flow cytometry

Monoclonal antibodies against chicken monocyte/ macrophages (clone KUL01, isotype IgG_1,_ mouse anti-chicken monocyte/ macrophages-PE), CD4 (clone CT-4, isotype IgG_1,_ mouse anti-chicken CD4-PE), CD8 (clone CT-8, isotype IgG_1,_ mouse anti-chicken CD8α-FITC) and CD3 (clone CT-3, isotype IgG_1_) were purchased from Southern Biotechnology (Birmingham, Ala, USA). Mouse anti-chicken CD40 (clone AV79, isotype IgG2a) and mouse anti-chicken MHC II monoclonal (clone 21–1A6, isotype IgG_1_) antibodies were used as primary antibodies (purchased from Bio-Rad, Raleigh, NC, USA). PerCP/Cy5.5 goat anti-mouse IgG secondary antibody and isotype control were purchased from Bio Legend (San Diego, CA, USA).

## Experimental design

### Mucosal delivery of CpG-ODN via the intrapulmonary route

Synthetic CpG**-**ODN was diluted in sterile, distilled water and using a Compressor Nebulizer(705–470) unit (AMG Medical Inc; Montreal, QC, Canada), mucosal delivery of CpG-ODN was performed as aerosolized micro-droplets (particle size of 0.5–5 µm) in a closed 0.036 m^3^ acrylic chamber containing 40 birds/chamber for 15 min (4 mg CpG-ODN/ chamber) at hatch. The control group of birds was aerosolized with sterile saline for 15 min in the acrylic chamber using a similar compressor nebulizer. Each group had 80 birds (n = 80). The temperature was maintained at 28–30 °C in the acrylic chamber during the administration of CpG-ODN or saline. The CpG-ODN delivery technique and the duration of nebulization is based on our previous published^27^ and unpublished data to facilitate uniform distribution of CpG-ODN in chicks.

### *E. coli* challenge

Two days post IPL delivery of CpG-ODN (day-2 post-hatch), birds were challenged with either 1 × 10^5^(n = 20/group) or 1 × 10^6^ cfu (n = 20/group) of a virulent strain of *E. Coli* by subcutaneous injection in the neck, following the previosuly published challenge model^27^. Data were collected on mortality, clinical signs, pathology and bacterial isolations from the air sacs for 7 days following challenge with *E. coli*.

### Sample collection

QuantiGene Plex assay: Spleen and lung samples from three chicks per group were collected at 10-time points post treatment (0, 3, 6, 12, 24, 32, 48, 72 hr, day 5 and day 7) in 1.5 mL tubes, flash frozen in dry ice and ethanol slurry and stored in −80 °C.

### Flow cytometry

Four chicks from each group were humanely euthanized at 24, 48 and 72 hr post treatment by cervical dislocation. Spleen and lung tissues were collected into 1.5 mL tubes, rinsed with PBS and kept on ice until processed on the same day.

### Analysis of cytokine gene expression

Expression of mRNA of IFN-α, IL-18, IFN-γ, IL-1β, LITAF, IL-4, IL-6, andIL-10 cytokine genes in the lung and spleen were measured by commercially available probes for avian cytokines QuantiGene Plex 2.0® (Panomics/Affymetrix Inc., Fremont, CA, USA). The genes of interest and their accession numbers are listed in Table 1.

**Table 1:** Genes of interest.

Gene abbreviation	Gene name	Accession number
Hprt 1*	Hypoxanthine-guanine phosphoribosyl transferase 1	NM_204848
IFN-α	Interferon alpha	NM_205427
IFN-γ	Interferon gamma	NM_205149
IL-1β	Interleukin 1, beta	NM_204524
IL-4	Interleukin 4	NM_001007079
IL-6	Interleukin 6	NM_204628
IL-10	Interleukin 10	NM_001004414
IL-18	Interleukin 18	NM_204608
LITAF	Lipopolysaccharide-induced TNF factor	NM_204267
Tubb 1*	Tubulin, beta 1	NM_205445

(* housekeeping genes- HKGs).

Frozen spleen and lung tissues were processed, and tissue homogenates were prepared following the manufacturer’s instructions with some modifications. Briefly, whole organ was chopped in small pieces using scalpel and 5 mg of chopped tissue was homogenized in 300 µL of homogenization solution containing 3 µL of Proteinase K. The tissue lysate was digested at 65 °C to release the mRNA and centrifuged to precipitate the debris. The supernatants of tissue lysates were collected and saved at −80 °C for future use. The oligonucleotide capture probes for mRNA and label probes were designed by the manufacturer. The tissue homogenates (40 µL/well) were added to a 96-well plate that was pre-loaded with 210 µL of the capture reagent per well and the respective probe set. After overnight hybridization at 54 ± 1 °C, further processing was carried out according to the manufacturer’s instructions by hybridizing with bDNA pre-amplifier 2.0, bDNA amplifier 2.0, biotinylated probe and substrate. Luminescence was measured using a Luminex instrument (Bio-Rad, USA). Signals recorded as the mean luminescence intensity generated from each bead are proportional to the amount of each mRNA captured on the surface of each generated specific probe set^33^. The expressions of these genes were normalized with the expression of housekeeping genes (HKGs), tubulin beta 1 (Tubb1) and Hypoxanthine-guanine phosphoribosyl transferase 1 (Hprt 1). For analysis, MFI data of each gene was divided with the average HKGs (Hprt 1 and Tubb 1) to normalize the data. Then, using normalized MFI data fold expression of CpG-ODN group in comparison to saline control group at each time point was calculated.

### Flow cytometry

Cell populations isolated from lung and spleen were stained with cell marker specific antibodies to identify the presence of monocyte/macrophages, CD4^+^, and CD8^+^ T cells. The monocyte/macrophages were further analysed for the expression of maturation markers (CD40 and MHCII). Briefly, ~5 × 10^5^ cells were incubated with mouse anti-chicken monocyte/macrophage (PE) antibody at 4 °C for 30 min for detecting antigen presenting cells (APCs). Cells from the previous step were washed three times and incubated separately with either mouse anti-chicken CD40 or MHCII primary antibodies at 4 °C for 30 min to identify maturation markers on the APCs. After three washings with PBS, the cells were stained with PerCP/Cy5.5 goat anti-mouse IgG secondary antibody at 4 °C for 30 min. In order to detect the ratio of CD4^+^ and CD8^+^ T cells, another set of ~5 × 10^5^ cells were incubated with mouse anti-chicken CD8 (FITC), CD4 (PE), and CD3 (SPRD) together at 4 °C for 30 min. Finally, the washed cells were suspended in 300 µL of PBS in glass flow cytometry tubes and subjected to flow cytometry analysis using the Epics XL (Beckman Coulter) and FACS Caliber (BD Bioscience). Acquired data were analyzed with FlowJo software (Tree Star, Inc., Ashland, OR, USA).

### Immunohistochemistry

The spleen and lung samples from the CpG-ODN and control groups were fixed in 4% buffered paraformaldehyde for over 24 hr and then embedded in paraffin. The samples were sectioned at a 5 μm thickness and fixed on poly-L-lysine coated slides for the immunohistochemical staining. Briefly, the slides were subjected to deparaffinization and washing in PBS. The endogenous peroxidase activity of tissue sections were inactivated by covering them with 3% hydrogen peroxide for 10 min. The slides were blocked with 5% bovine serum albumin and then incubated with the primary anti-chicken CD3 antibodies at a 1:200 dilution (clone CT-3, Southern Biotech, USA) at 4 °C overnight. Following the PBS wash, the slides were incubated with a secondary reagents and DAB (3,3'Diaminobenzidine).The development of dark brown color indicated a positive reaction.

### Statistical analysis

The significance of difference in survival analysis, bacteriological scoring, CCS, difference of gene expression and cell populations between groups were analyzed using Prism (Prism 6.0, GraphPad Software Inc; San Diego, CA, USA) with a significance level of P < 0.05. The survival data of both 1 × 10^5^ cfu/bird and 1 × 10^6^ cfu/bird of *E. coli* were combined for clarity of analysis and presentation. The significance of differences among groups in survival patterns, median survival times and relative risk of mortality were analyzed using the log-rank test and chi-square statistics. Clinical scores of each bird for the 10 d period were summed to generate a CCS and the significance of differences among groups was tested using Kruskal Wallis nonparametric analysis of variance. Sidak multiple comparisons test following Ordinary One Way ANOVA was used to test the significant differences of gene expression between CpG-ODN group and the control group at each time point. The percentages of CD4^+^ and CD8^+^ T lymphocyte subsets were combined to compare the total T cell infiltration in the lung and spleen between groups. Difference of cellular infiltration and monocyte/macrophages maturation marker expression (MFI) between the two groups were compared using Student-t test with Mann-Whitney test.

## Results

### Immunoprotective effect of mucosal delivery of CpG-ODN against E. coli septicemia

**Figure 1:**
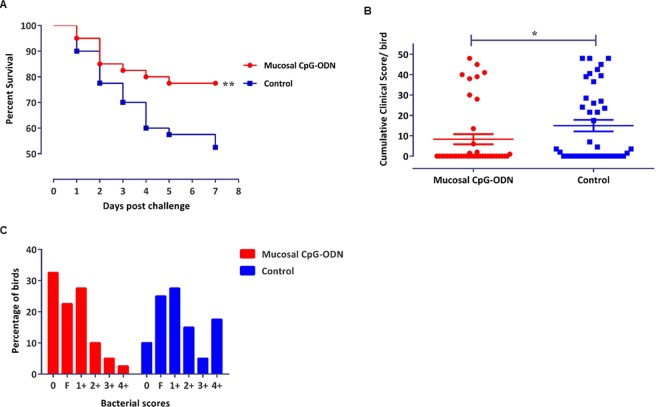
Survival percentages, CCS and bacterial scores of the birds that received mucosal CpG-ODN (n = 40) and/or saline control (n = 40) and followed by a lethal *E. coli* challenge. (**A**) Birds that received CpG-ODN showed significantly higher survival compared to the saline control group (P = 0.0249). (**B**) CCS values following the *E. coli* challenge depicted significantly lower CCS in the CpG-ODN received group compared to the saline control (P = 0.0384). (**C**) Birds following 1 × 10^5^ CFU or 1 × 10^6^ CFU/bird *E. coli* challenge indicated a heavier bacterial load in the saline control birds compared to the IPL CpG-ODN treated birds.

During the seven days post *E. coli* challenge, the group that received mucosal CpG-ODN as micro droplets through the intrapulmonary route showed a significantly higher survival (P = 0.0249) compared to the saline control group (Fig. 1A). The cumulative clinical scores (CCS) following *E. coli* challenge with 1 × 10^5^ or 1 × 10^6^ cfu/bird exhibited that the group that received CpG-ODN through mucosal route had a significantly lower CCS (P = 0.0384) compared to the control group (Fig. 1B). Bacterial isolation from the thoracic air sacs tended to show a higher bacterial load in the control chicks compared to the CpG-ODN group (Fig. 1C). The relative risk of mortality following the *E. coli* challenge was reduced by 53.92% following CpG-ODN treatment.

### Analysis of cytokine gene expression

**Figure 2:**
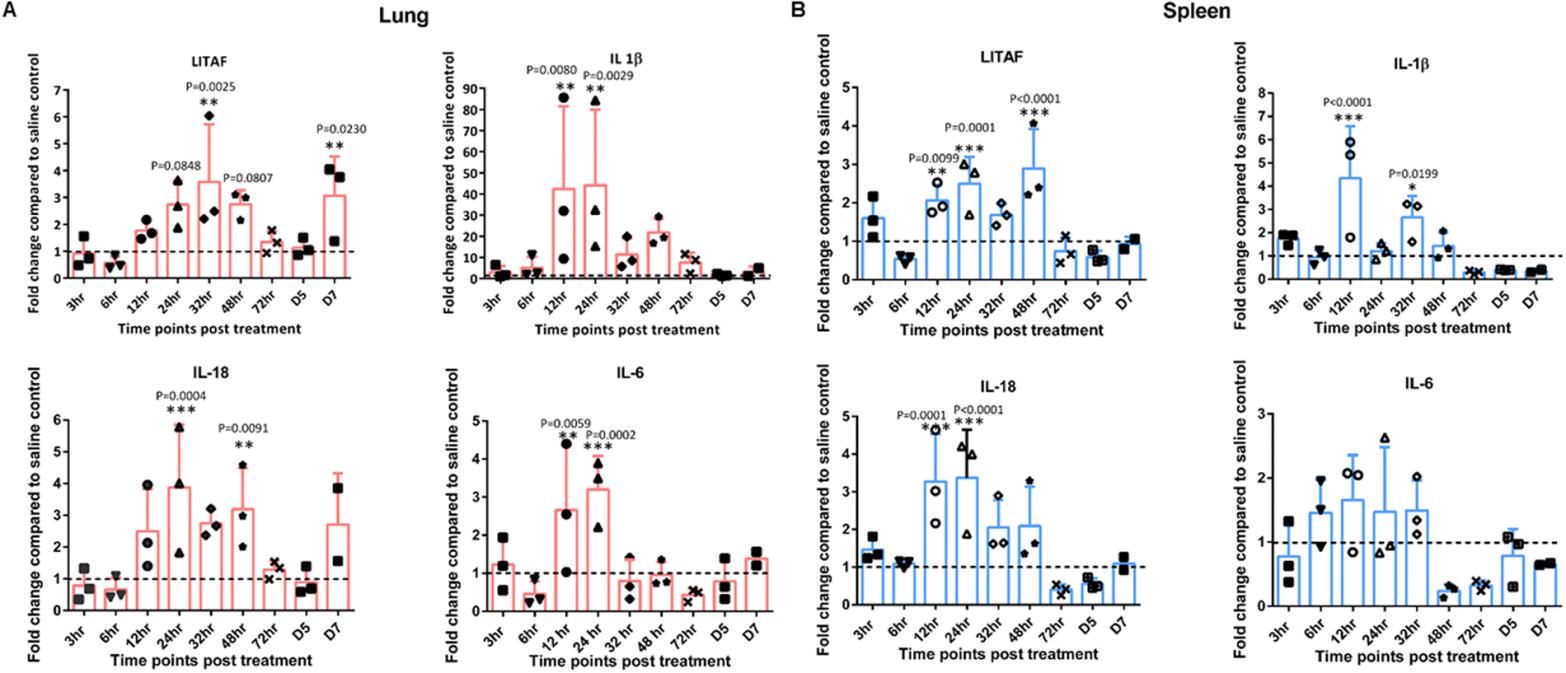
Pro-Inflammatory cytokine profiles in lungs and spleen. The luminescence intensity of each gene was normalized to the averages of HKGs (Hprt 1 and Tubb 1). Fold changes were calculated against the control at each time point. (Broken lines indicate the fold change of one, which shows no change compared to the control group). Sidak multiple comparisons test following ANOVA was used to analyse the significance of gene expression between CpG-ODN and the saline control group at each time point. Asterisks indicate the fold changes that were significantly different (P < 0.05).

**Figure 3:**
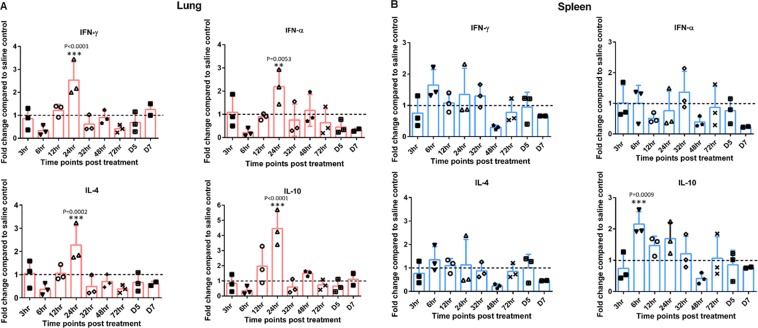
Interferon and regulatory cytokine profile in lung and spleen. The luminescence intensity of each gene was normalized to the averages of HKGs (Hprt 1 and Tubb 1). Fold changes were calculated against the control at each time point. (Broken lines indicate the fold change of one, which shows no change compared to the control group). Sidak multiple comparisons test following ANOVA was used to analyse the significance of gene expression between CpG-ODN and the saline control group at each time point. Asterisks indicate the fold changes that were significantly different (P < 0.05).

CpG-ODN mediated immune modulation was elucidated by measuring the expression array of pro-inflammatory (IL-1β, IL-6, LITAF, IL-18), Th-1 type (IFN-α, IFN-γ and Th-2 type (IL-4, IL-10) cytokines in spleen and lung at various time points post mucosal CpG-ODN delivery. It was identified that the fold changes of mRNA levels were 1 or >1 almost all times, in the chicks that received IPL CpG-ODN compared to the control group. There was no specific consistent pattern of gene expression; however, pro-inflammatory cytokine genes (IL-1β, IL-6, LITAF, and IL-18) exhibited relatively higher levels in the mucosal CpG-ODN group compared to the control. Level of expression of IL-1β gene was significantly many folds higher in the lung compared to that of the spleen starting from 6 through 72 hr peaking at 12 and 24 hr. LITAF expression increased 24 to 48 hr post treatment in both lung and spleen whereas IL-18 elevated from 12 to 48 hr (Fig. 2). Both Th-1 type cytokine genes (IFN-α, IFN-γ and Th-2 type cytokines (IL-4, IL-10) were generally highly expressed in the lung compared to spleen in the mucosal CpG-ODN treated birds, peaking at 24 hr post treatment (Fig. 3).

### Flow cytometry analysis and histology

Flow cytometry analysis revealed that mucosal delivery of CpG-ODN via the intrapulmonary route significantly influenced the infiltration of antigen presenting cells (APCs) such as monocytes/macrophages with mature phenotypes as well as the T cell populations in the lungs and spleen. The supplementary figure 1 shows the flow cytometry gating strategy to identify monocytes/macrophages in the spleen (A) and the lung (B), respectively. The supplementary figure 2 represents the gating strategy followed to investigate CD4^+^CD3^+^ and CD8^+^CD3^+^ T cells in the spleen (supplementary figure 2A) and the lung (supplementary figure 2B).

### Immune cell infiltration in the spleen and lung tissues following CpG-ODN mucosal delivery

**Figure 4:**
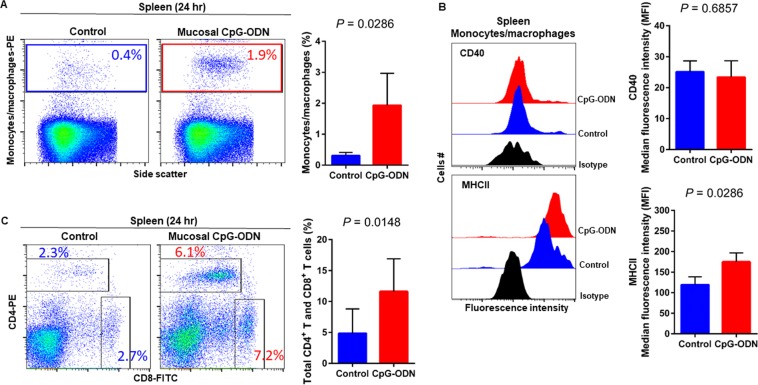
Flow cytometric analysis of spleen cells at 24 hr post IPL CpG-ODN or saline treatment. (**A**) Macrophage analysis was performed by gating on monocytes/macrophages population in the spleen based on forward and side scatter. Bar diagram represents percent monocytes/macrophages infiltration. (**B**) Histogram and bar diagram displays the median fluorescence intensity (MFI) of CD40 (upper panel) and MHC II (lower panel) expression on monocytes/macrophages, respectively. Cells were gated as shown in the panel A. (**C**) T lymphocyte population in the spleen analysed by gating on the lymphocyte population based on forward and side scatter plot. Quantification of CD4^+^ and CD8^+^ T lymphocytes was done by using PE-labeled mouse anti-chicken CD4 and FITC-labeled mouse anti-chicken CD8 monoclonal antibodies. Bar diagram indicates the mean total CD4^+^ plus CD8^+^ T lymphocyte percentages in the spleen following IPL CpG-ODN treatment. Difference of cellular infiltration and monocyte/macrophages maturation marker expression (MFI) between the CpG-ODN administered group vs saline control group were compared using Student-t test with Mann-Whitney test, with P < 0.05 being statistically significant. Error bar = Standard Deviation (n = 4).

**Figure 5:**
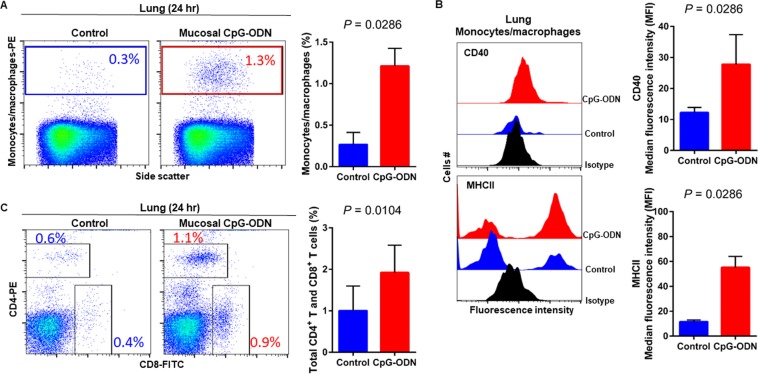
Flow cytometric analysis of lung cells at 24 hr  post IPL CpG-ODN or saline treatment. (**A**) Macrophage analysis was performed by gating on monocytes/macrophages population in lungs based on forward and side scatter. Bar diagram represents percent monocytes/macrophages infiltration. (**B**) Histogram and bar diagram displays the median fluorescence intensity (MFI) of CD40 (upper panel) and MHC II (lower panel) expression on monocytes/macrophages, respectively. Cells were gated as shown in the panel A. (**C**) T lymphocyte population in the lung analysed by gating on the lymphocyte population based on forward and side scatter plot. Quantification of CD4^+^ and CD8^+^ T lymphocytes was done by using PE-labeled mouse anti-chicken CD4 and FITC-labeled mouse anti-chicken CD8 monoclonal antibodies. Bar diagram indicates the mean total CD4^+^ plus CD8^+^ T lymphocyte percentages in the lung following IPL CpG-ODN treatment. Difference of cellular infiltration and monocyte/macrophages maturation marker expression (MFI) between the CpG-ODN administered group vs saline control group were compared using Student-t test with Mann-Whitney test, with P < 0.05 being statistically significant. Error bar = Standard Deviation (n = 4).

**Figure 6:**
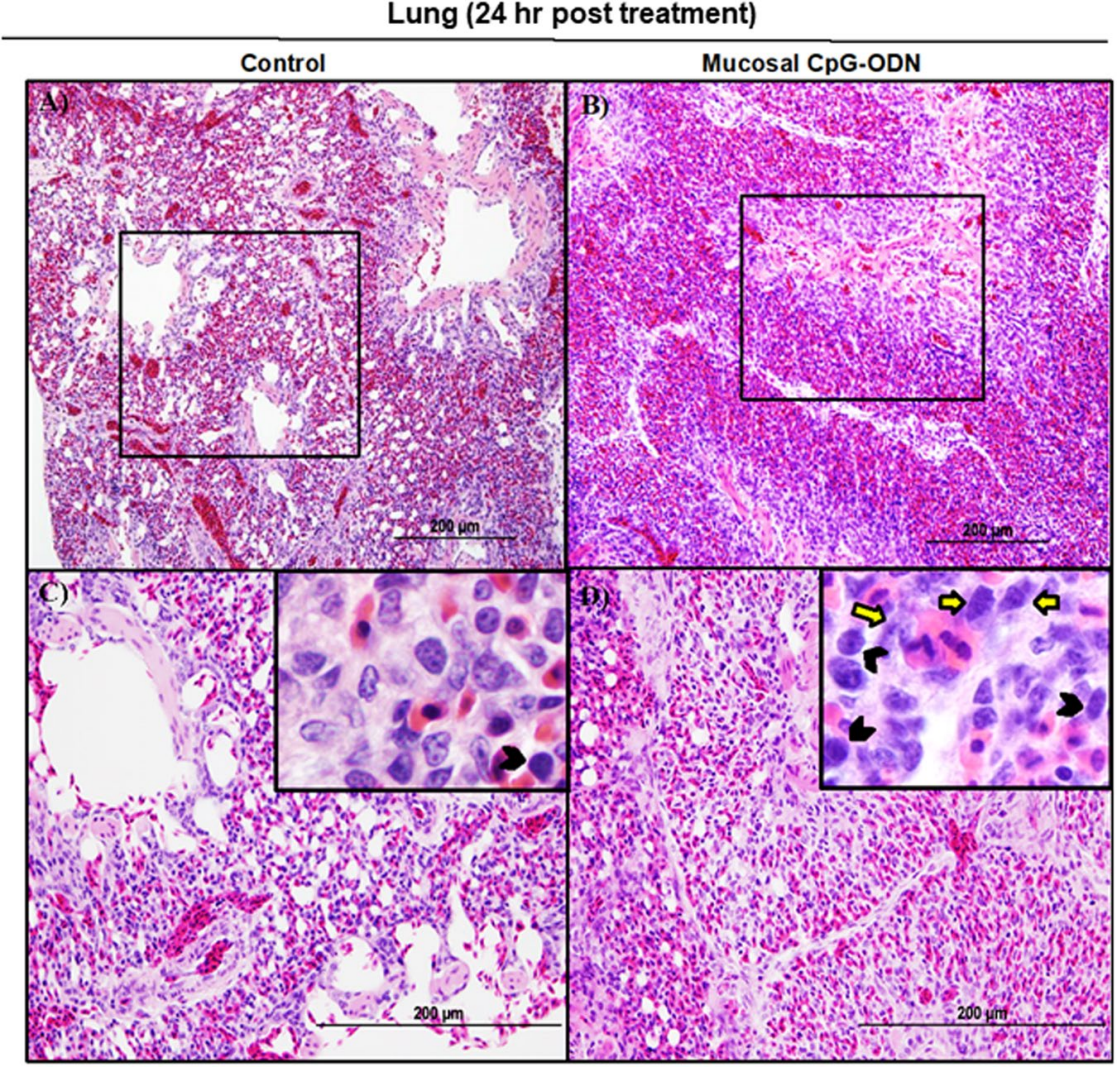
Histology of lungs 24 hr post treatment with IPL saline (**A** and **C**) or IPL CpG-ODN (B and D). Low power images (20×) indicated a clearly visible high degree of immune cell infiltration in the CpG-ODN treated lung (**B**) compared to the less cellularity and clearly visible air spaces in the saline control lungs (**A**). At higher power (40× and 100×), the IPL CpG-ODN received lung showed a distinctly higher number of lymphocytes (black arrow heads in the insert) and large mononuclear cells resembling monocytes/ macrophages (yellow arrows in the insert) infiltrated into the parenchyma (**D**) compared to the less number of those cells visible in the saline control lung (**C**).

**Figure 7:**
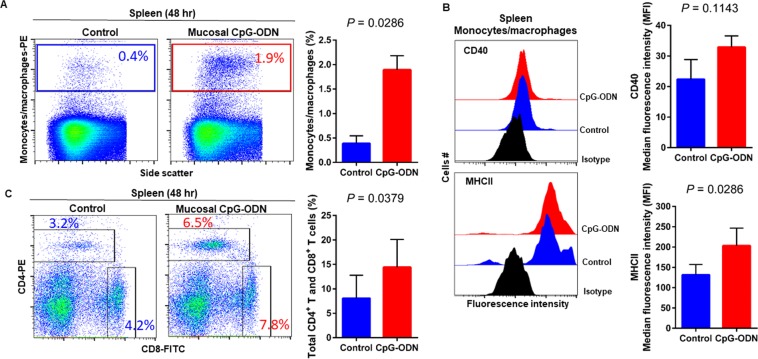
Flow cytometric analysis of spleen cells at 48 hr post IPL CpG-ODN or saline treatment. (**A**) Macrophage analysis was performed by gating on monocytes/macrophages population in the spleen based on forward and side scatter. Bar diagram represents percent monocytes/macrophages infiltration. (**B**) Histogram and bar diagram displays the median fluorescence intensity (MFI) of CD40 (upper panel) and MHC II (lower panel) expression on monocytes/macrophages, respectively. Cells were gated as shown in the panel A. (**C**) T lymphocyte population in the spleen analysed by gating on the lymphocyte population based on forward and side scatter plot. Quantification of CD4^+^ and CD8^+^ T lymphocytes was done by using PE-labeled mouse anti-chicken CD4 and FITC-labeled mouse anti-chicken CD8 monoclonal antibodies. Bar diagram indicates the mean total CD4^+^ plus CD8^+^ T lymphocyte percentages in the spleen following IPL CpG-ODN treatment. Difference of cellular infiltration and monocyte/macrophages maturation marker expression (MFI) between the CpG-ODN administered group vs saline control group were compared using Student-t test with Mann-Whitney test, with P < 0.05 being statistically significant. Error bar = Standard Deviation (n = 4).

**Figure 8:**
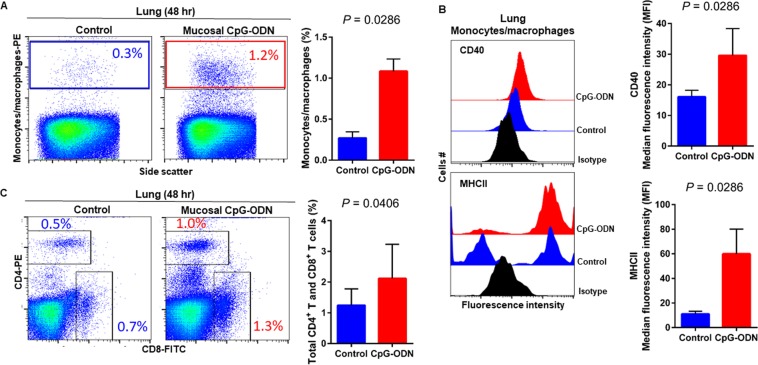
Flow cytometric analysis of lung cells at 48 hr post IPL CpG-ODN or saline treatment. (**A**) Macrophage analysis was performed by gating on monocytes/macrophages population in lungs based on forward and side scatter. Bar diagram represents percent monocytes/macrophages infiltration. (**B**) Histogram and bar diagram displays the median fluorescence intensity (MFI) of CD40 (upper panel) and MHC II (lower panel) expression on monocytes/macrophages, respectively. Cells were gated as shown in the panel A. (**C**) T lymphocyte population in the lung analyzed by gating on the lymphocyte population based on forward and side scatter plot. Quantification of CD4^+^ and CD8^+^ T lymphocytes was done by using PE-labeled mouse anti-chicken CD4 and FITC-labeled mouse anti-chicken CD8 monoclonal antibodies. Bar diagram indicates the mean total CD4^+^ plus CD8^+^ T lymphocyte percentages in the lung following IPL CpG-ODN treatment. Difference of cellular infiltration and monocyte/macrophages maturation marker expression (MFI) between the CpG-ODN administered group vs saline control group were compared using Student-t test with Mann-Whitney test, with P < 0.05 being statistically significant. Error bar = Standard Deviation (n = 4).

**Figure 9:**
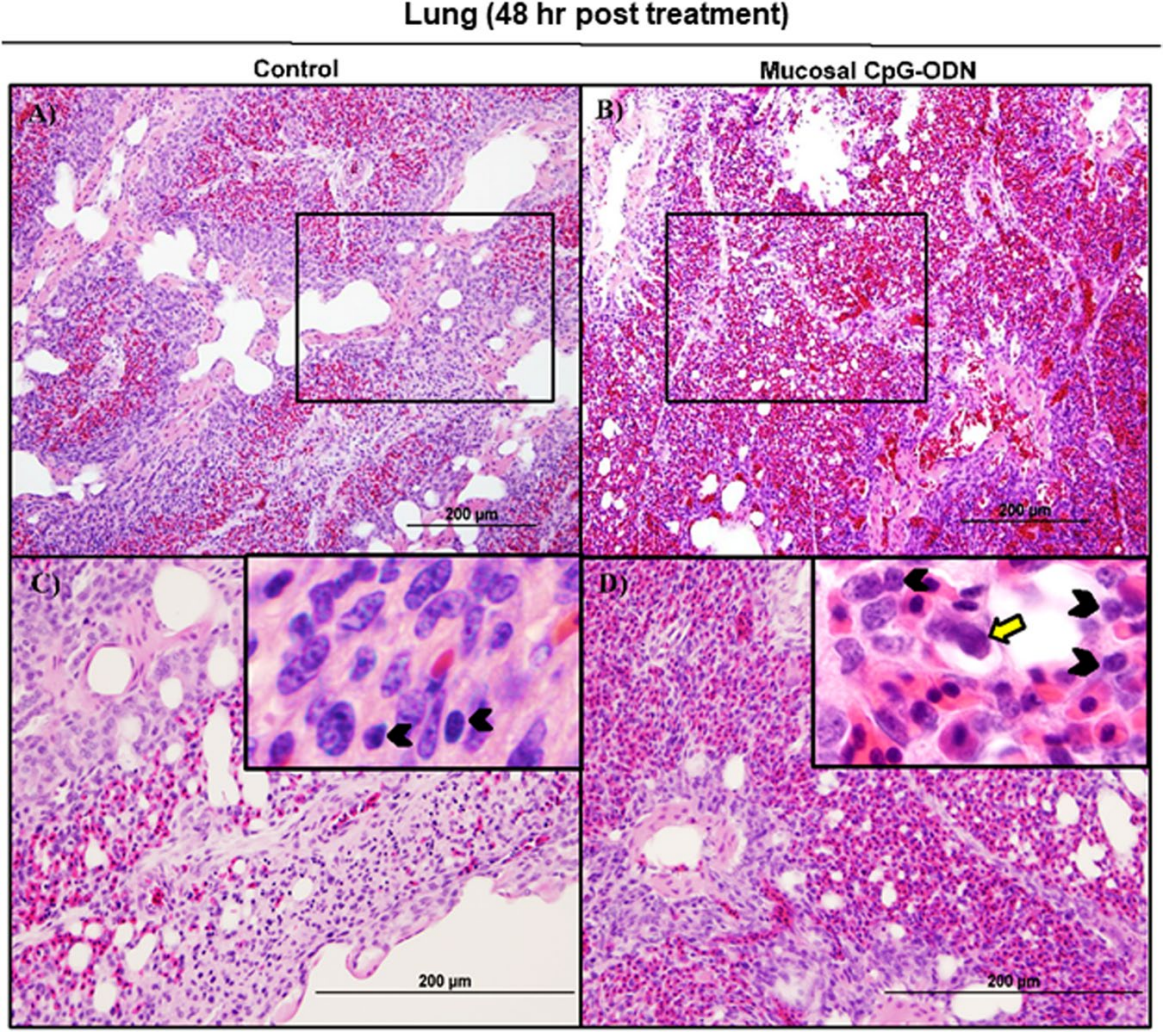
Histology of the lungs 48 hr following IPL saline (**A** and **C**) and IPL CpG-ODN delivery (**B** and **D**). At lower magnification (20×), saline control lungs appeared less cellular and paler with clear air spaces (**A**) than the CpG-ODN treated lungs distinctly infiltrated with large numbers of lymphocytes (**B**). However, this cellularity in the CpG-ODN lungs was visibly quite lower than what was observed 24 hours post treatment. At higher magnification (40× and 100×), more lymphocytes (black arrowheads in the insert) and mononuclear cells resembling monocytes and macrophages (yellow arrows in the insert) were seen in the parenchyma of IPL CpG-ODN lungs (**D**) compared to the saline control lungs (**C**).

**Figure 10:**
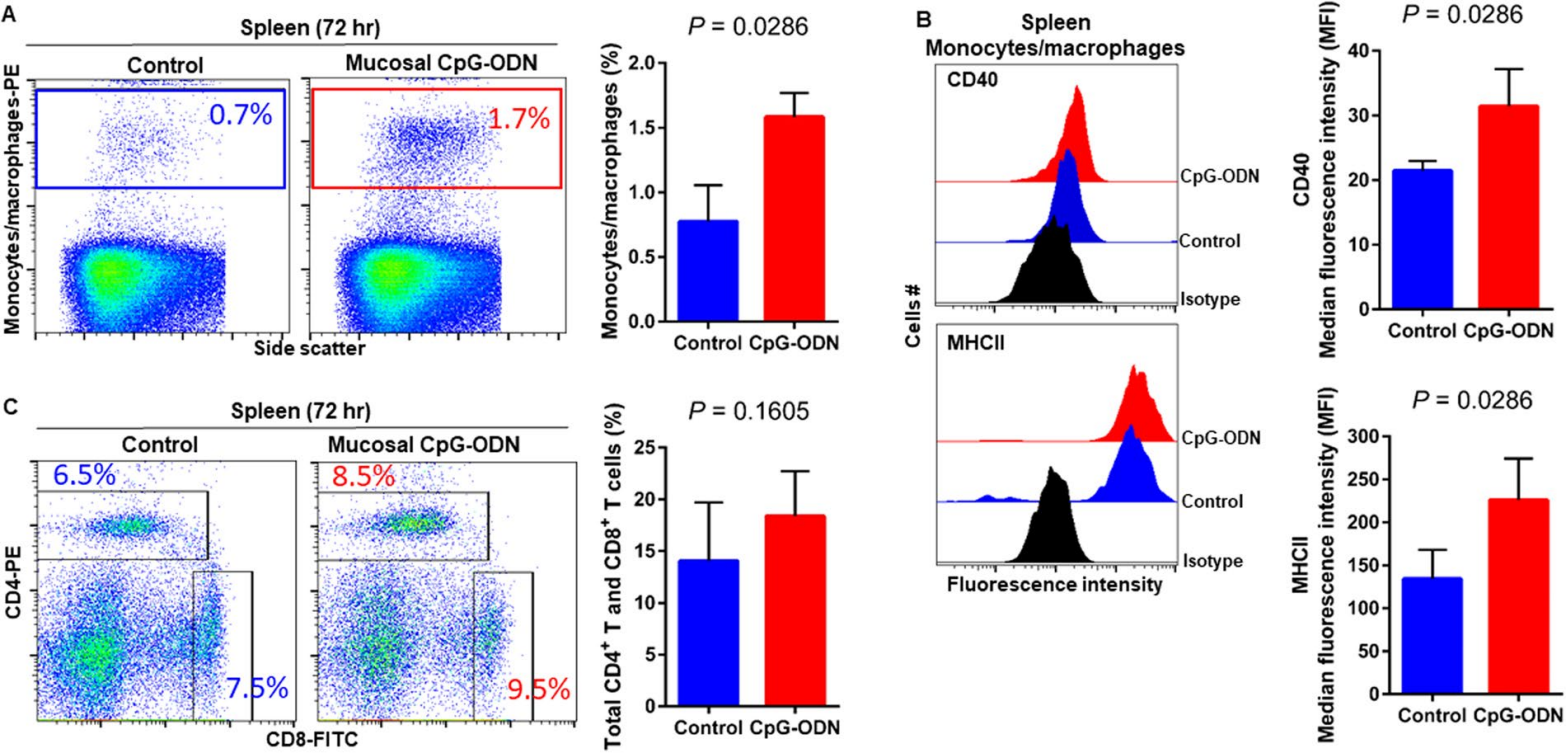
Flow cytometric analysis of spleen cells at 72 hr post IPL CpG-ODN or saline treatment. (**A**) Macrophage analysis was performed by gating on monocytes/macrophages population in the spleen based on forward and side scatter. Bar diagram represents percent monocytes/macrophages infiltration. (**B**) Histogram and bar diagram displays the median fluorescence intensity (MFI) of CD40 (upper panel) and MHC II (lower panel) expression on monocytes/macrophages, respectively. Cells were gated as shown in the panel A. (**C**) T lymphocyte population in the spleen analysed by gating on the lymphocyte population based on forward and side scatter plot. Quantification of CD4^+^ and CD8^+^ T lymphocytes was done by using PE-labeled mouse anti-chicken CD4 and FITC-labeled mouse anti-chicken CD8 monoclonal antibodies. Bar diagram indicates the mean total CD4^+^ plus CD8^+^ T lymphocyte percentages in the spleen following IPL CpG-ODN treatment. Difference of cellular infiltration and monocyte/macrophages maturation marker expression (MFI) between the CpG-ODN administered group vs saline control group were compared using Student-t test with Mann-Whitney test, with P < 0.05 being statistically significant. Error bar = Standard Deviation (n = 4).

**Figure 11:**
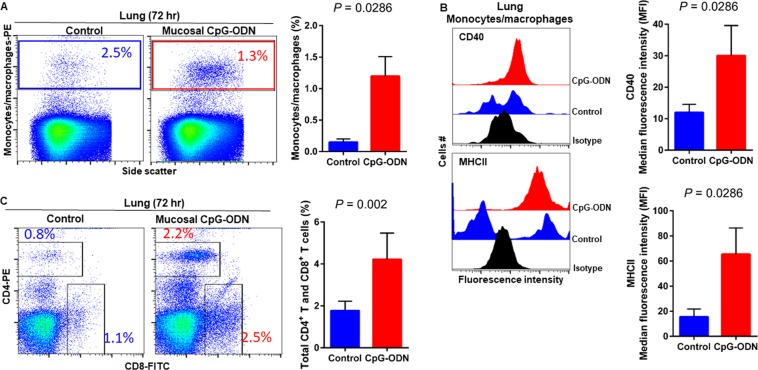
Flow cytometric analysis of lung cells at 72 hr post IPL CpG-ODN or saline treatment. (**A**) Macrophage analysis was performed by gating on monocytes/macrophages population in lungs based on forward and side scatter. Bar diagram represents percent monocytes/macrophages infiltration. (**B**) Histogram and bar diagram displays the median fluorescence intensity (MFI) of CD40 (upper panel) and MHC II (lower panel) expression on monocytes/macrophages, respectively. Cells were gated as shown in the panel A. (**C**) T lymphocyte population in the lung analyzed by gating on the lymphocyte population based on forward and side scatter plot. Quantification of CD4^+^ and CD8^+^ T lymphocytes was done by using PE-labeled mouse anti-chicken CD4 and FITC-labeled mouse anti-chicken CD8 monoclonal antibodies. Bar diagram indicates the mean total CD4^+^ plus CD8^+^ T lymphocyte percentages in the lung following IPL CpG-ODN treatment. Difference of cellular infiltration and monocyte/macrophages maturation marker expression (MFI) between the CpG-ODN administered group vs saline control group were compared using Student-t test with Mann-Whitney test, with P < 0.05 being statistically significant. Error bar = Standard Deviation (n = 4).

**Figure 12:**
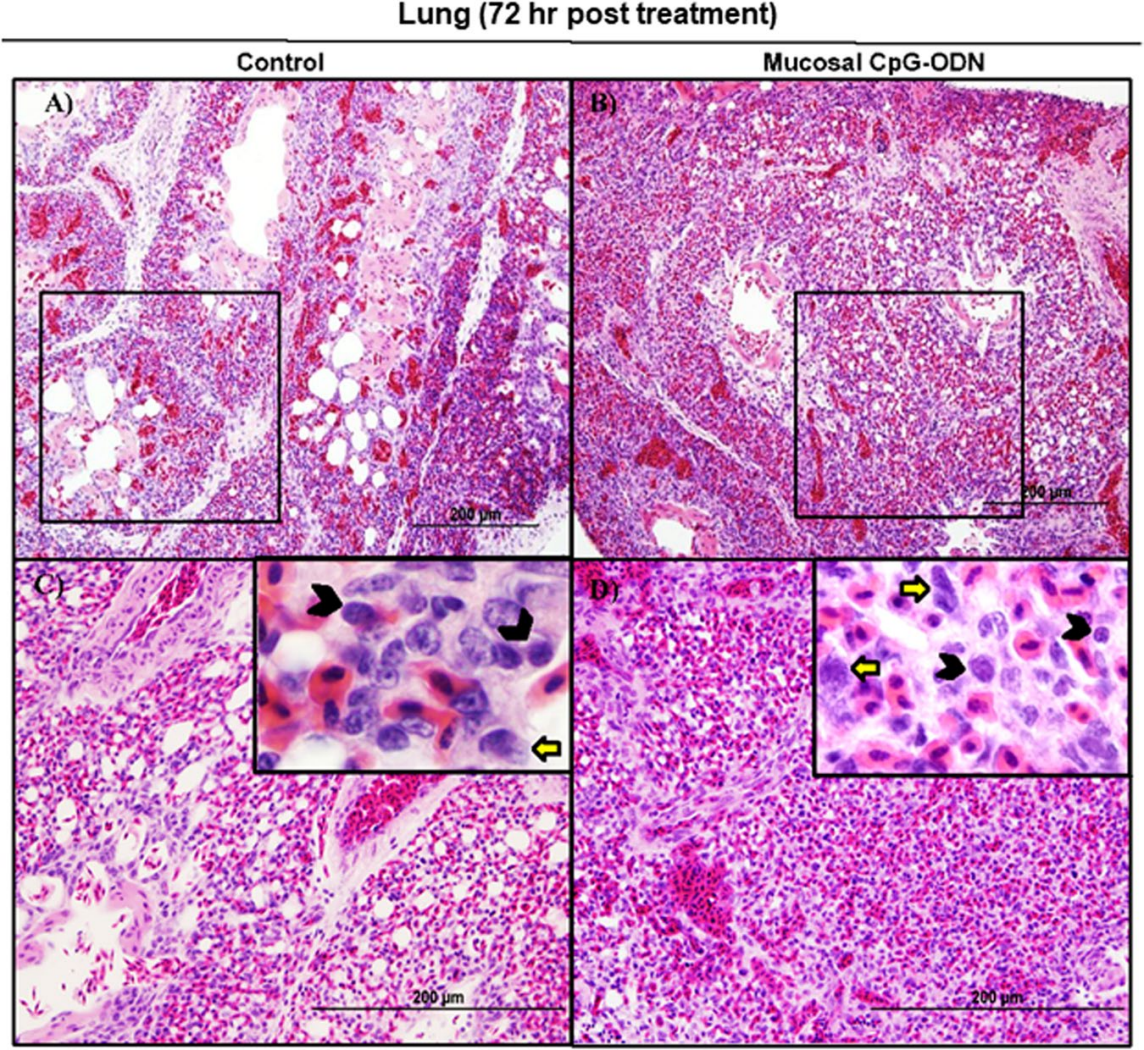
Histology of lungs 72 hr following IPL saline (**A** and **C**) and IPL CpG-ODN (**B** and **D**) delivery. At lower magnification (20×), the saline control lung showed slightly less cellularity and more air spaces (**A**) compared to the CpG-ODN treated lung appearing compact and more cellular (**B**), although the contrast was less striking 72 hours post treatment. At higher magnification (40× and 100×), the IPL CpG-ODN treated lung (**D**) contained visibly more lymphocytes (black arrow heads in the insert) and large mononuclear cells resembling monocytes/ macrophages (yellow arrows in the insert) compared to the saline control lungs (**C**).

**Figure 13:**
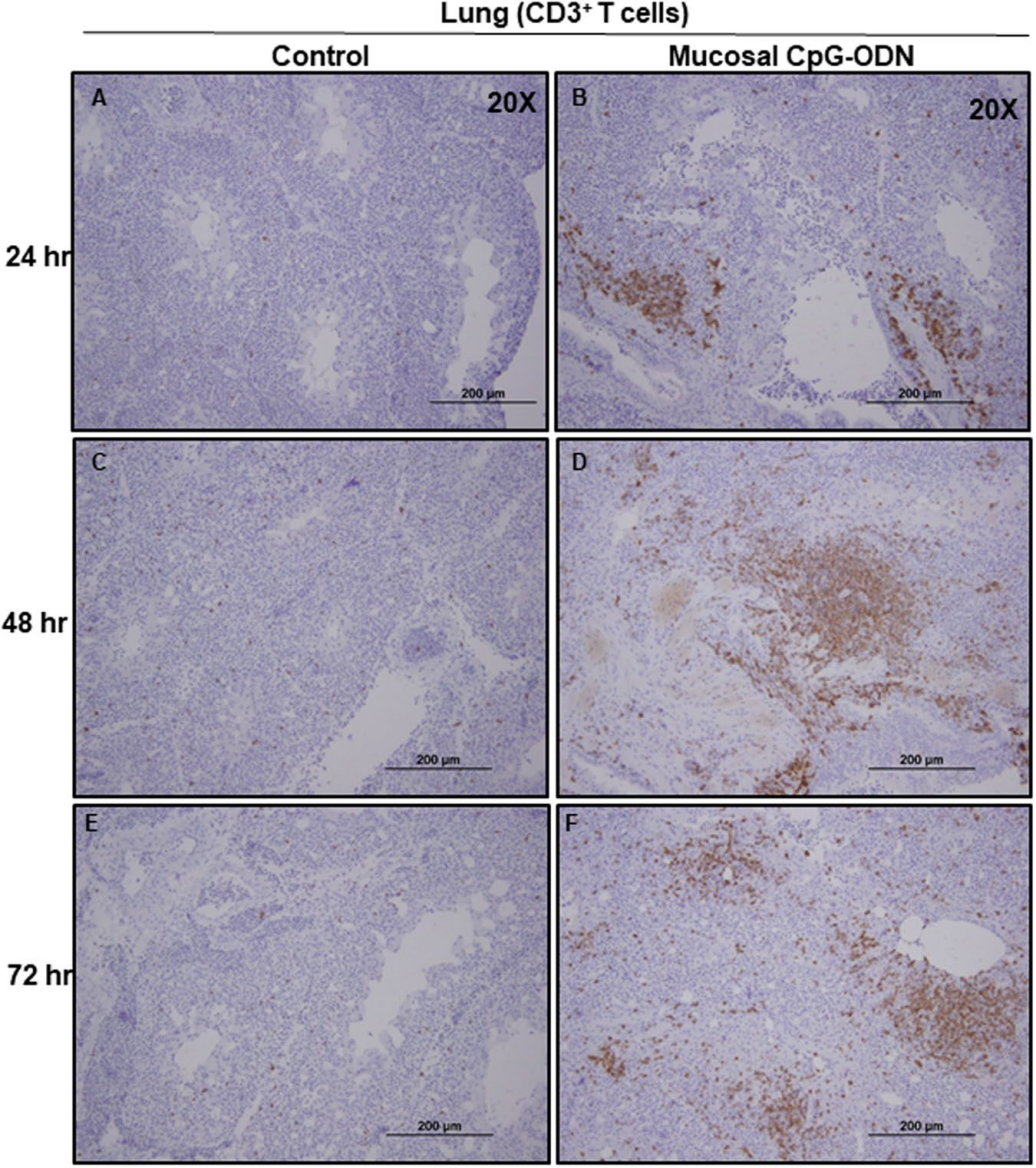
Immunohistochemistry of lungs 24, 48, and 72 hrs following IPL saline (**A**,**C**, and **E**) and IPL CpG-ODN (**B**,**D**, and **F**) delivery, respectively. At magnification 20×, the CpG-ODN treated lung showed substantially high infiltration of CD3^+^ T cells (brown color) compared to the saline control lung at 24 hr (**B** vs. **A**), 48 hr (**D** vs. **C**), and 72 hr (**F** vs. **E**) post treatment.

**Figure 14:**
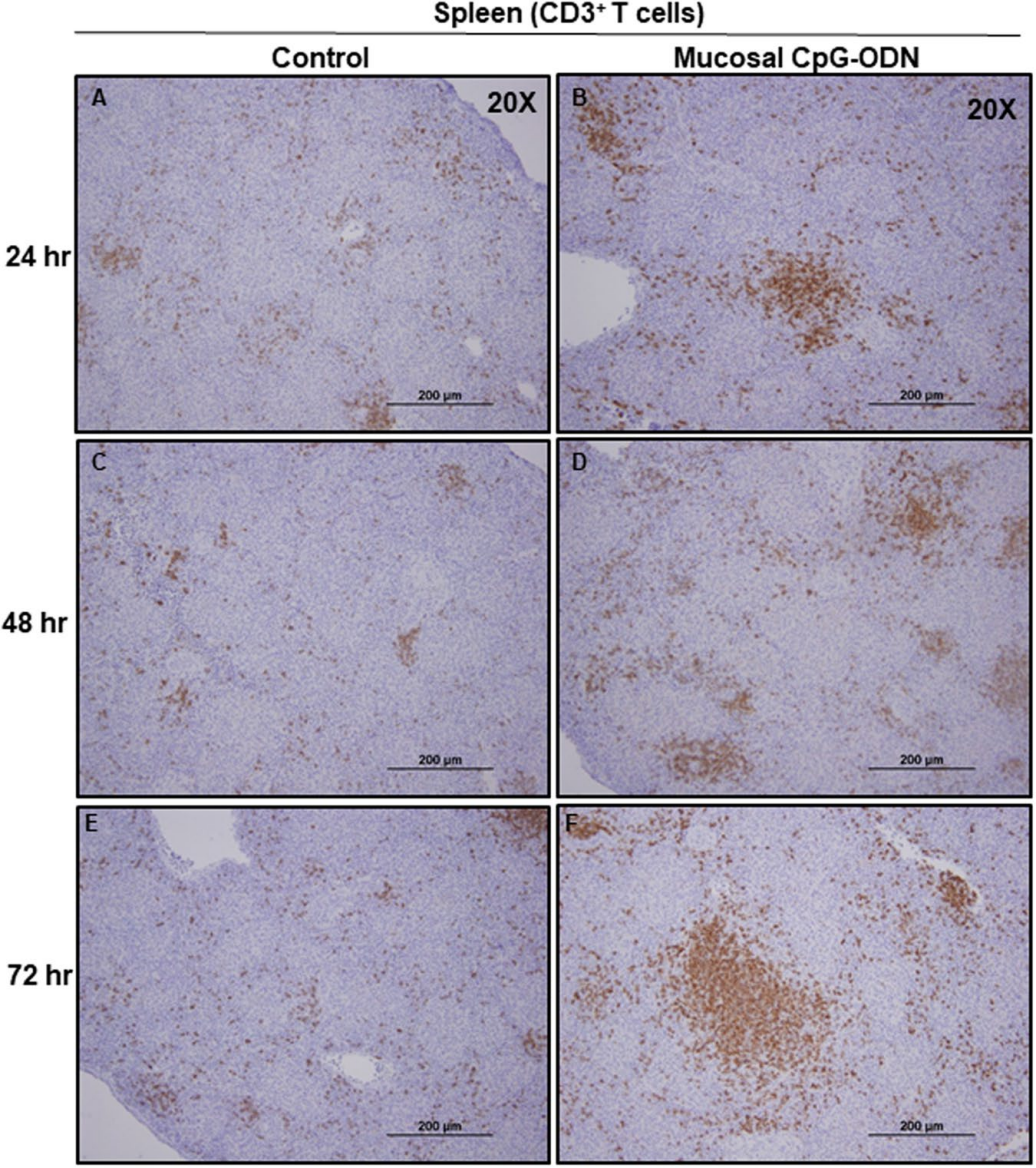
Immunohistochemistry of spleen 24, 48, and 72 hrs following IPL saline (**A**,**C**, and **E**) and IPL CpG-ODN (**B**,**D**, and **F**) delivery, respectively. At magnification 20×, the CpG-ODN treated spleen revealed substantially high infiltration of CD3^+^ T cells (brown color) compared to the saline control lung at 24 hr (**B** vs. **A**), 48 hr (**D** vs. **C**), and 72 hr (**F** vs. **E**) post treatment.

Flow cytometry data at 24 hr post CpG-ODN treatment revealed a significant infiltration of monocytes/macrophages in the spleen (Fig. 4A). We did not observe an upregulation of costimulatory molecule CD40 in these spleen-infiltrating monocytes/macrophages (Fig. 4B, upper panel). However, the expression of antigen presenting molecule MHCII by these infiltrating splenic monocytes/macrophages was significantly higher in CpG-ODN group at 24 hr (Fig. 4B, lower panels). The combined percentage of CD4^+^ and CD8^+^ T cell subsets were markedly increased in spleen of CpG-ODN treated chicks compared to the control (Fig. 4C). Flow cytometry data of the lung showed significantly greater infiltration of monocytes/macrophages (Fig. 5A). Moreover, CD40 (Fig. 5B, upper panel) and MHCII (Fig. 5B, lower panel) was highly upregulated in the lung-infiltrating monocytes/macrophages indicating maturation of these APCs. Also in the lung, the combined percentage of CD4^+^ and CD8^+^ T cell subsets were significantly higher in the CpG-ODN group (Fig. 5C). The histological examination of the lung showed very high infiltrations of monocytes/macrophages and lymphocytes in CpG-ODN group (Fig. 6B,D) compared to control (Fig. 6A,C). At 48 hr post CpG-ODN delivery, flow cytometry showed increased infiltration of monocytes/macrophages in the spleen (Fig. 7A). However, like the 24 hr time-point, we did not find a significant increase in CD40 expression by infiltrating splenic monocytes/macrophages at the 48 hr time-point (Fig. 7B, upper panel). But, the expression of MHCII by spleen infiltrating macrophages was significantly higher in CpG-ODN group at 48 hr (Fig. 7B, lower panels). At the 48 hr time point, the combined percentage of CD4^+^ and CD8^+^ T cell subsets distinctly increased in spleen of CpG-ODN treated chicks (Fig. 7C). In the lung, monocytes/macrophages at the 48 hr time-point was substantially high (Fig. 8A) as well as the level of CD40 (Fig. 8B, upper panel) and MHCII (Fig. 8B, lower panel) expression was also significant. The lung showed significantly high infiltration of T cell subsets (CD4^+^ and CD8^+^ T cell combined) 48 hr after CpG-ODN treatment (Fig. 8C). The histological examination also supports higher infiltration of monocytes/macrophages and lymphocytes in the lungs of CpG-ODN group (Fig. 9B,D) compared to control (Fig. 9A,C). At the 72 hr time point, flow cytometry revealed not only greater infiltration of monocytes/macrophages (Fig. 10A) but also significantly upregulated costimulatory molecule CD40 (Fig. 10B, upper panel) and antigen presenting molecule MHC II (Fig. 10B, lower panel) in the splenic monocytes/macrophages. This data indicated that mucosal delivery of CpG-ODN via the intrapulmonary route is able to induce maturation of monocytes/macrophages in the spleen, though with some delay. At 72 hr post CpG-ODN treatment infiltration of T cell subsets (CD4^+^ and CD8^+^ T cell combined) in the spleen appeared higher in the CpG-ODN group but it was not statistically different than control (Fig. 10C). Flow cytometry data from lung tissues at 72 hr post CpG-ODN delivery demonstrated significantly greater infiltrations of monocytes/macrophages (Fig. 11A). The expression of costimulatory molecule CD40 and antigen presenting molecule MHCII by these infiltrating monocytes/macrophages was significantly higher in CpG-ODN group (Fig. 11B, upper and lower panels, respectively) at 72 hr, providing strong evidence of enhanced maturation of these sentinel immune cells (Fig. 11B). The MHC negative cells observed in our flow cytometry analysis could be monocytes^34^ or immature macrophages. Flow cytometry of lung tissues at the 72 hr time-point demonstrated markedly higher infiltration of T cell subsets (CD4^+^ and CD8^+^ T cell combined) in the CpG-ODN group (Fig. 11C). The histological examination of lung showed higher infiltrations of immune cells in the CpG-ODN group (Fig. 12B,D) compared to control (Fig. 12A,C). The immunohistochemistry analysis of lung (Fig. 13) and spleen (Fig. 14) at 24, 48, and 72 hrs post-CpG-ODN administration revealed marked infiltration of CD3^+^ T cells in CpG-ODN group compared to saline control. Although, our immunohistochemical staining for CD3^+^ T cells worked on the formalin fixed lung and spleen samples, but it did not work for macrophage detection on formalin fixed samples (data not shown). Altogether, CpG-ODN mucosal delivery was able to accelerate immunological development in neonatal broilers by orchestrating the enrichment of immunological niches in the spleen and the lung of neonatal chicks. However, studies involving many other immune subsets are warranted.

## Discussion

The lung is a major site of entry for bacterial and viral pathogens in the chicken. We recently used mucosal delivery of aerosolized CpG-ODN alone through intrapulmonary route, found that the antibacterial protection kicks off as soon as 6 hr post mucosal delivery of CpG-ODN and remains effective at least for 5 days in neonatal chicks^27^. Also in this study, we found significant protection in chicks following mucosal delivery of CpG-ODN indicating the effectiveness of mucosal route in inducing protective immunity against bacterial infections. Our mucosal delivery of CpG-ODN directly targeted the lung. Therefore, the current study was designed to understand the mechanisms by which the mucosal delivery of aerosolized CpG-ODN alone provides protection against *E. coli* septicemia. We were interested in investigating cytokine responses and cellular infiltration levels at the site of CpG-ODN treatment that is the lung, to understand the local immune modulatory effects and also the spleen as a secondary immune organ to examine the systemic immune response.

When a bacterial pathogen enters the body, pathogens are sensed by specific pattern recognition receptors, predominantly TLRs, which recognize PAMPs such as lipopolysaccharides, flagellin, and unmethylated CpG motifs. This recognition leads to the secretion of pro-inflammatory cytokines like IL-1β and IL-6 as well as chemokines that attract phagocytic heterophils and macrophages to the site of infections as a response^35^. Since synthetic CpG-ODNs mimic bacterial unmethylated CpG motifs,similar innate and adaptive downstream mechanisms are expected. Using *in vitro* experiments, He *et al*. showed that CpG-ODN stimulates significant amounts of nitric oxide (NO) by chicken monocytes^23^ and pro-inflammatory cytokine mRNA IL-1β and IL-6. At the same time, considerable induction of chemokine mRNA such as IL-8 and MIP-1α within 2 hr post-treatment was observed in chicken monocytes indicating their potential role in trafficking leukocytes to infected sites^36^. Previously, Patel *et al*. demonstrated that *in ovo* delivered CpG-ODN induced predominantly a Th1 type of immune response in the spleens of chicks^25^. However, a previous report^37^ and our study^26^ demonstrated both Th1 and Th2 types of cytokines in chickens in after CpG-ODN treatment.

In the present study, we explored the expression levels of 8 cytokine genes; pro-inflammatory cytokines (IL-1β, IL-6, LITAF, IL-18), Th1 type (IFN-γ, IFN-α) and Th2type (IL-4, IL-10) in order to understand the pathways of anti-bacterial effects following mucosal delivery of CpG-ODN *via* intrapulmonary route. For exploring the cytokine mRNA expression, we used QuantiGene Plex 2.0® assay technique; a multiplex assay that measures the expression levels of multiple cytokine genes in a single well using probe hybridization technique and without PCR amplification. Our results indicated that all cytokines noticeably increased in both lungs and spleens following CpG-ODN administration. In our study, we observed upregulation of both Th1 and Th2 cytokines in the lungs and spleens following mucosal delivery of CpG-ODN. This observation is in agreement with our recent data that demonstrated both Th1 and Th2 cytokine expression following *in ovo* delivery of CpG-ODN in eighteen days old embryonated eggs^26^. Another study also supports the finding that CpG-ODN induces both Th1 and Th2 types of cytokines in chickens^37^. Type II IFNs (IFN-γ) is an important cytokine in the Th1 type immune response and it plays a vital role in controlling viral and intracellular bacterial infections. IFN-γactivates macrophages and thereby increases phagocytosis and the production of potent antimicrobial products such as (NO), oxygen free radicals and hydrogen peroxide. IFN-γ also helps naïve CD4^+^and CD8^+^T lymphocytes to become Th1 and effector cells to combat pathogens in cell-mediated immunity^38^. Other studies using *in ovo* delivery of CpG-ODN in chickens indicated elevated levels of both IFN-γ and IFN-α in the spleens^25,39^. In our study, we observed a statistically significant increase of IFN-γ and IFN-α levels particularly in the lungs within 24 hr of treatment as well as noticeably higher levels in the spleens following mucosal delivery of CpG-ODN. The statistically elevated levels of cytokine gene expressions in the lung could be due to the direct delivery of CpG-ODN in lungs.

Cytokines IL-1β, IL-6, and IL-12 together with TNF-α act synergistically to eliminate the infection^40^. Our study indicated that mucosal delivery of CpG-ODN promoted the upregulation of pro-inflammatory cytokine genes; IL-1β, IL-6, LITAF, and IL-18 significantly in the lung and spleen. Overall, the cytokine gene expression was significantly higher in the lungs compared to the spleens, which may suggest that CpG-ODN is a potent mucosal immune stimulator when delivered through the intrapulmonary route. Present results support our recent study^26^ and other reports of CpG-ODN-mediated induction of pro-inflammatory cytokine genes in various organs^39,41,42^ as well as in avian thrombocytes^43^. We observed a remarkable upregulation of IL-1β mRNA expression, which was many folds higher than the expression of other cytokine genes. IL-1β is an important pro-inflammatory cytokine secreted largely by macrophages as well as NK cells, B-lymphocytes, dendritic cells, fibroblasts, and epithelial cells. IL-1β initiates macrophage activation and NO production^44^ and contributes to inflammation by increasing vascular permeability and expressing endothelial adhesion molecules^45^.

Furthermore, IL-1β is known to stimulate the release of other pro-inflammatory cytokines such as IL-6 and TNF-α^45^. IL-6 is well known for being synthesized during acute infection and providing immune protection via synthesis of acute phase proteins, hematopoiesis, and differentiation of naïve T lymphocytes^46^. Elevation of IL-6 mRNA expression within the first 24 hr post-CpG-ODN treatment in our study, particularly in the lungs, suggests its contribution to the immunological defence against *E. coli* infection. We observed a statistically significant elevation of IL-18 mRNA gene expression in both lungs and the spleen of the CpG-ODN treated birds. IL-18 mainly induces IFN-γ production from T cells and NK cells which is vital in antimicrobial defence. Chicken LITAF is primarily secreted by macrophages^47^. It is noteworthy that LITAF and IL-18 remained upregulated in both lungs and spleen for 48 hr post-CpG-ODN treatment. Importantly, among all the cytokines tested here, LITAF and IL-18 expression again upregulated at day 7 post-treatment in lungs. It was reported earlier that IL-18 promotes monocytes TNF-α production^48^. TNF-α is a multifunctional cytokine that induces upregulation of CD40 and other costimulatory molecules in antigen presenting cells such as macrophages and dendritic cells^49^. In agreement with our recent study that used *in ovo* (embryo injection) delivery of CpG-ODN, the present data using mucosal delivery of CpG-ODN also highlights the importance of LITAF in CpG-ODN-induced immunoprotective mechanisms.

We next explored the recruitment kinetics of immune cells using flow cytometry in the non-lymphoid target organ, that is lung, as well as in the lymphoid organ, spleen, following mucosal delivery of CpG-ODN via intrapulmonary route. Data revealed a significant increase in the number of monocytes/macrophages in the lungs and spleen at all time-points studied (24–72 hr). At the same time, we examined the kinetics of T lymphocytes in the spleen and lungs. We observed a significantly increased level of both CD4^+^ and CD8^+^ T lymphocytes in both lungs and spleen (except at the 72 hr time point in spleen) after mucosal delivery of CpG-ODN. The histological examination of the lung showed increased cellularity with monocytes/macrophages and lymphocytes infiltration following CpG-ODN mucosal delivery. These flow cytometry and histological data strongly suggested the enrichment of these organs as immunological niches.

The occurrence of costimulatory molecules, such as CD40, CD80, and CD86, is a sign of macrophage activation and maturation^50^. CD40 signalling is involved in the maturation of APCs such as macrophages and dendritic cells^51^. Dendritic cells licensing via CD40 signaling facilitates CD8^+^ T-cell priming^52^ thereby induces protective CD8^+^ cytotoxic T cell (CTL) immunity^53^. In our recent study, we found upregulation of CD40 but not CD80 or CD86 in monocytes/macrophages following *in ovo* administration of CpG-ODN^26^. Therefore, in this study, we investigated the expression of CD40 on monocytes/macrophages in spleens and lungs of CpG-ODN treated and control groups. We found significant upregulation of CD40 in monocytes/macrophages in the lungs throughout our study (24–72 hr). In contrast, CD40 expression in the splenic monocytes/macrophages of control and CpG-ODN treated groups was not different, even after 48 hours post CpG-ODN mucosal delivery. Nonetheless, at the 72 hr, CD40 was significantly upregulated in the splenic monocytes/macrophages of CpG-ODN group. These data suggest that although monocytes/macrophages infiltration in spleen significantly increased throughout our study (24–72 hr), monocytes/macrophages maturation, as evidenced by CD40 expression, was delayed in the spleen. The differences in CD40 expression kinetics between lungs and spleen could be explained by the fact that CpG-ODN was directly delivered to lung mucosa. Interestingly, these flow cytometry and cytokine data indicate that mucosal delivery of CpG-ODN micro-droplets via intrapulmonary route activates targeted tissue as well as influences distant secondary organs such as the spleen, demonstrating a systemic effect. The lung is the most common site of entry for a majority of pathogens of young chicks before they disseminate in the body^54^. The number of macrophages in the lungs of control chicks was significantly low, which may partly explains why these young chicks are so susceptible to infections during early neonatal life. A significant increase in the number of macrophages in the lungs of chicks after CpG-ODN exposure suggests that these young chicks are better equipped with sentinel cells to combat pathogenic insults in the barn environment. Thus, increasing the availability of antigen presenting cells in the lung through mucosal delivery of CpG-ODN plays a vital role in inducing antibacterial immunity.

A previous study reported that a major development of the avian spleen begins after hatching with the exposure to various antigens^55^. Our data showed that mucosal delivery of CpG-ODN stimulated and created immunological niches in the lung and the spleen thus accelerating the immune development. In a human phase I trial, intramuscular injection of CpG-ODN caused infiltration of T lymphocytes at the injection site wherein the secretion of chemokines by the activated APCs provided chemoattractant to bring more lymphocytes to the site^56^. Our recent data^26^ and a study by De Silva *et al*. reported the infiltration of CD4^+^ and CD8^+^ T lymphocytes into the lungs following *in ovo* delivery of CpG-ODNs^57^. We found rapid and significant upregulation of LITAF in lungs and spleens that can also potentially induce infiltration of immune cells, which is also supported by a previous study showing TNF-α promotes infiltration of lymphocytes and other immune cells to the site of inflammation^44^. A previous study reported that TNF-α stimulates MHCII expression in macrophages^58^. Our finding of enhanced MHCII expression in monocytes/macrophages in the lungs and concurrent significantly increased LITAF expression in the lungs suggest important roles of these cytokines in CpG-ODN-mediated antibacterial immunity.

In conclusion, the enhanced immune cell enrichment and multifunctional cytokine expression augment immunocompetence in CpG-ODN-treated neonatal chicks. Our study has demonstrated for the first time that mucosal delivery of CpG-ODN micro-droplets via the intrapulmonary route accelerates immunological development by enriching immune niches not only in the target organ (lungs) but also at distant organs such as the spleen.

## Supplementary information

Supplementary information.
