# Supplementary material for: Mucosal delivery of CpG-ODN mimicking bacterial DNA via the intrapulmonary route induces systemic antimicrobial immune responses in neonatal chicks
Source: Sci Rep. 2020 Mar 24;10:5343. doi: 10.1038/s41598-020-61683-y (PMC7093454; doi:10.1038/s41598-020-61683-y)
Supplement: Supplementary file 1 — Supplementary information. [file 41598_2020_61683_MOESM1_ESM.pdf]

Supplementary information for:

Mucosal delivery of CpG-ODN mimicking bacterial DNA via the intrapulmonary route induces systemic antimicrobial immune responses in neonatal chicks

Kalhari Goonewardene[1], Khawaja Ashfaque Ahmed[1]*, Thushari Gunawardana[1], Shelly Popowich[1], Shanika Kurukulasuriya[1], Ruwani Karunarathna[1], Ashish Gupta[1], Lisanework E. Ayalew[1], Betty Lockerbie[1], Marianna Foldvari[2], Suresh Tikoo[3], Philip Willson[4], and Susantha Gomis[1]*

[1]Department of Veterinary Pathology, Western College of Veterinary Medicine, University of Saskatchewan, Saskatoon, SK, S7N 5B4, Canada.
[2]School of Pharmacy, University of Waterloo, 200 University Avenue West, Waterloo, ON, N2L 3G1, Canada.
[3]Vaccinology and Immunotherapy, School of Public Health, University of Saskatchewan, Saskatoon, SK, S7N 5E3, Canada
[4]Canadian Centre for Health and Safety in Agriculture, University of Saskatchewan, Saskatoon, SK S7N 2Z4 Canada

*Corresponding author mailing address: Department of Veterinary Pathology, Western College of Veterinary Medicine, 52 Campus Drive, University of Saskatchewan, Saskatoon, SK Canada S7N 5B4. Phone: (306) 966-7299. Fax: (306) 966-7439. Email: susantha.gomis@usask.ca (SG) kaa201@mail.usask.ca (KAA).

Supplementary figures

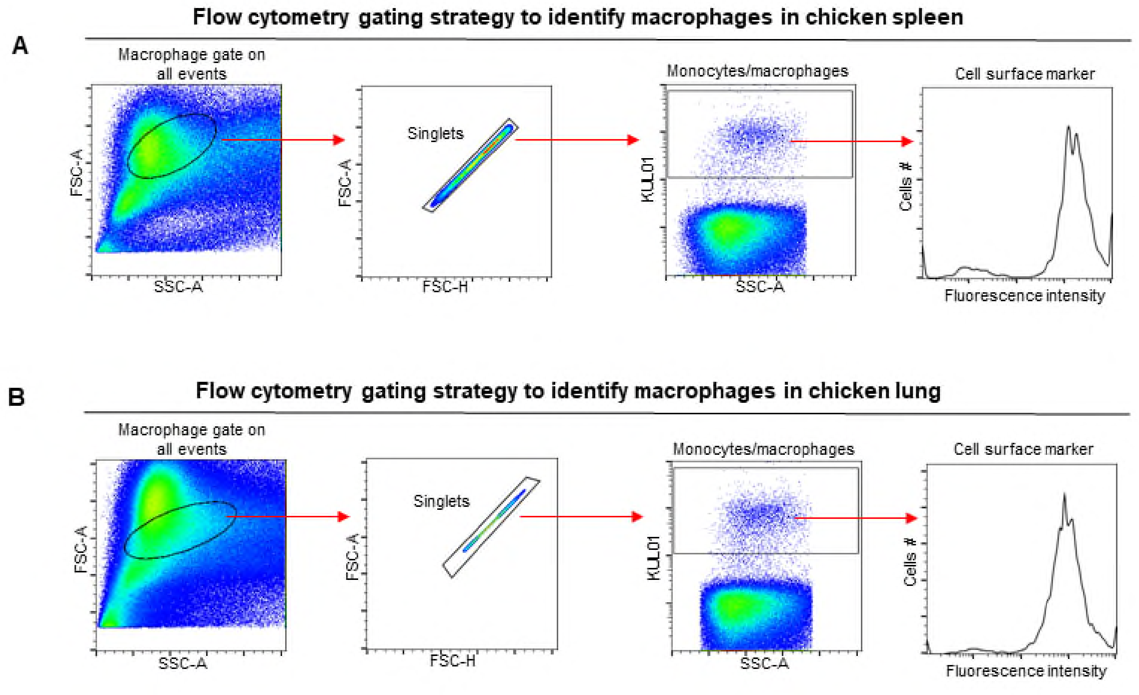

Supplementary figure 1: Gating strategy used to identify monocytes/macrophages in the chicken spleen and lung. Macrophage analysis was performed by gating on monocytes/macrophages population in the spleen (A), and lung (B) based on forward and side scatter applied on all events. Then, doublets were excluded from the analysis. On singlets, macrophages were identified using KUL01 staining (monocytes/macrophages marker), and side scatter. Finally, the histogram panel was generated gated on identified macrophages (KUL01+ cells) and cell surface molecules (CD40 or MHCII).

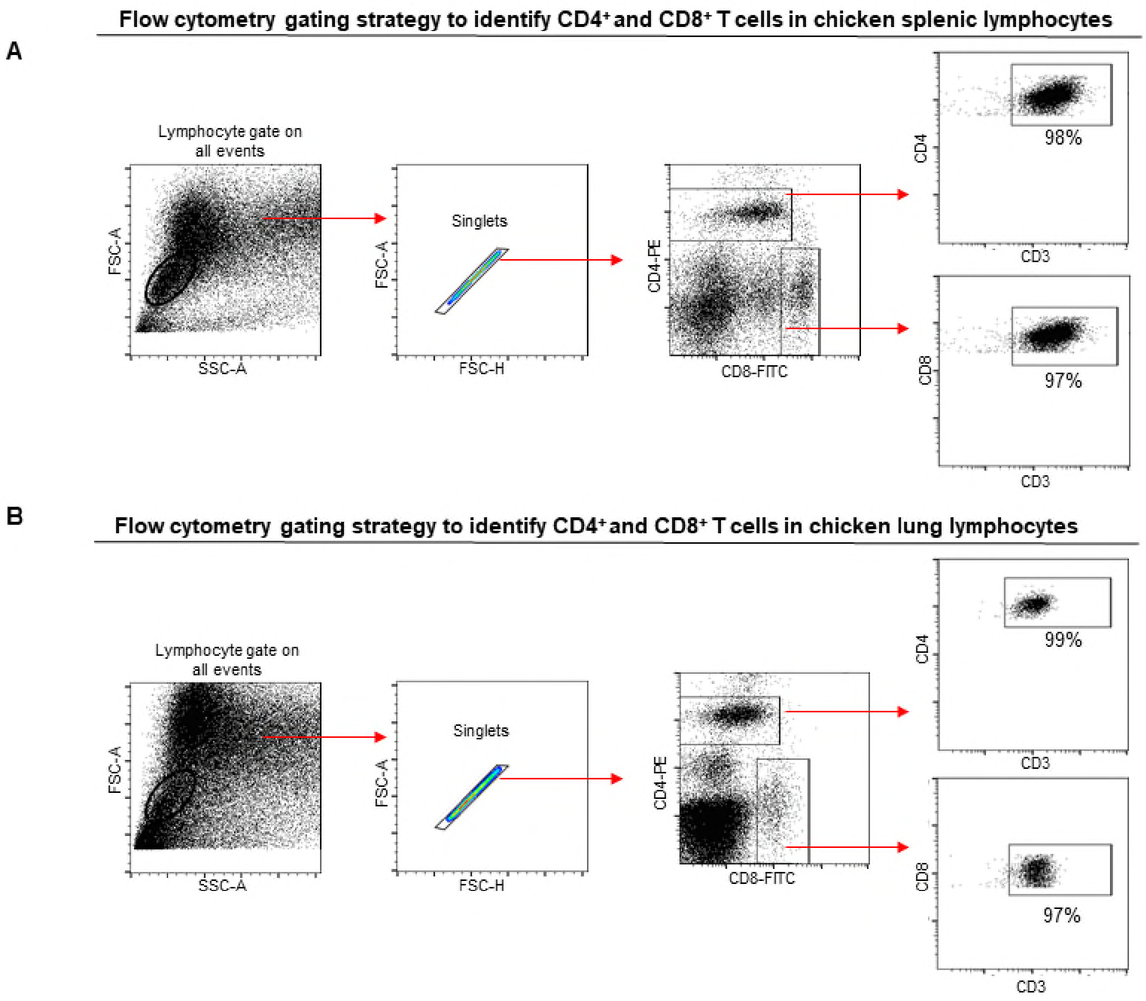

Supplementary figure 2: Gating strategy used to identify CD4$^+$ and CD8$^+$ T cells in the chicken spleen and lung. T cell subset analysis was performed by gating on the lymphocyte population on all events in the spleen (A), and lung (B) based on forward and side scatter. Then, doublets were excluded from the analysis. On singlets, CD4$^+$ and CD8$^+$ T cells were identified using anti-CD4 and anti-CD8 staining. Finally, the flow panel gated on CD4$^+$ and CD8$^+$ cell populations and CD3 staining revealed that >97% of the gated cells were T cells.
